# Gene functions of the *Ambystoma altamirani* skin microbiome vary across space and time but potential antifungal genes are widespread and prevalent

**DOI:** 10.1099/mgen.0.001181

**Published:** 2024-01-19

**Authors:** Emanuel Martínez-Ugalde, Víctor Ávila-Akerberg, Tanya M. González Martínez, Eria A. Rebollar

**Affiliations:** ^1^​ Centro de Ciencias Genómicas, Universidad Nacional Autónoma de México, Cuernavaca, Mexico; ^2^​ Instituto de Ciencias Agropecuarias y Rurales, Universidad Autónoma del Estado de México, Toluca, Mexico; ^3^​ Facultad de Ciencias, Universidad Nacional Autónoma de México, Ciudad de México, Mexico City, Mexico

**Keywords:** *Ambystoma altamirani*, Amphibian skin microbiome, Elevation, Functional genomic diversity, Seasonality

## Abstract

Amphibian skin microbiomes can play a critical role in host survival against emerging diseases by protecting their host against pathogens. While a plethora of biotic and abiotic factors have been shown to influence the taxonomic diversity of amphibian skin microbiomes it remains unclear whether functional genomic diversity varies in response to temporal and environmental factors. Here we applied a metagenomic approach to evaluate whether seasonality, distinct elevations/sites, and pathogen presence influenced the functional genomic diversity of the *A. altamirani* skin microbiome. We obtained a gene catalogue of 92 107 nonredundant annotated genes and a set of 50 unique metagenome assembled genomes (MAGs). Our analysis showed that genes linked to general and potential antifungal traits significantly differed across seasons and sampling locations at different elevations. Moreover, we found that the functional genomic diversity of *A. altamirani* skin microbiome differed between *B*. *dendrobatidis* infected and not infected axolotls only during winter, suggesting an interaction between seasonality and pathogen infection. In addition, we identified the presence of genes and biosynthetic gene clusters (BGCs) linked to potential antifungal functions such as biofilm formation, quorum sensing, secretion systems, secondary metabolite biosynthesis, and chitin degradation. Interestingly genes linked to these potential antifungal traits were mainly identified in Burkholderiales and Chitinophagales MAGs. Overall, our results identified functional traits linked to potential antifungal functions in the *A. altamirani* skin microbiome regardless of variation in the functional diversity across seasons, elevations/sites, and pathogen presence. Our findings suggest that potential antifungal traits found in Burkholderiales and Chitinophagales taxa could be related to the capacity of *A. altamirani* to survive in the presence of Bd, although further experimental analyses are required to test this hypothesis.

## Abbreviations

AaSMGC, ambystoma altamirani skin microbiome gene catalog; AF, antifungal function; BCC, bacterial competition and communication; Bd, batrachochytrium dendrobatidis; BGCs, biosynthetic gene clusters; COG, clusters of orthologous groups; GCPMR, genome copies per million reads; MAGs, metagenome assembled genomes; m.a.s.l, meters above sea level; NRPS, non-ribosomal peptides; ORFs, open reading frames; PCoA, principal coordinate analysis; PERMANOVA, permutational analysis of variance; RiPPs, ribosomally synthesized and post-translationally modified peptides; TPM, transcripts per million.

## Data Summary

Sequencing data have been deposited in the National Centre for Biotechnology Information (NCBI), Sequence Read Archive (SRA) within the BioProject PRJNA1010953. Metagenome assembled genomes were also deposited in the NCBI WGS under the BioProject PRJNA1010953. BioSample accessions for each MAG are included in Table S12, available in the online version of this article (in File S1). Bioinformatic workflow is available at https://github.com/EmanuelMartinez-Ugalde/A.-altamirani-skin-microbiome-Gene-catalog-and-MAG-recovery.

### Impact Statement

The amphibian skin microbiome plays an essential role in protecting hosts against the lethal infectious disease called chytridiomycosis, caused by the fungus *Batrachochytrium dendrobatidis*. Here we describe the functional genomic diversity of the skin microbiome of the endangered axolotl *Ambystoma altamirani* and determine its variation across seasons, elevations/sites, and the presence of the fungal pathogen. Our work identified bacterial genomes that are prevalent across time and space and include a wide repertory of potential antifungal genes that could play a relevant role in protecting their hosts against emerging diseases like chytridiomycosis.

## Introduction

The recent emergence of infectious fungal diseases that threaten various wildlife species [1–5] has promoted the development of conservation strategies to ameliorate their impact on susceptible species [6–10]. Due to their contribution to host development, nutrition, and health [11, 12], microbiomes have emerged as a promising field to aid in the conservation of endangered species [13]. It has been shown that host-associated microbiomes can contribute to immune system maturation [14–17], or disease resistance against pathogens [18–22]. For example, bacteria present in amphibian and bat microbiomes can inhibit the growth of fungal pathogens that have caused severe population declines in both groups of vertebrates [3, 5, 23, 24].

Amphibians nowadays are considered the most vulnerable group of vertebrates due to habitat reduction, introduction of invasive species, and the emergence of infectious diseases such as chytridiomycosis [3, 25–27]. This disease is caused by the fungal pathogens *Batrachochytrium dendrobatidis* (Bd) [28] and *B. salamandrivorans* (Bsal) [29], and it has been linked to population declines of more than 500 amphibian species worldwide [3]. Nonetheless, it has been shown that some amphibians can control the growth of these pathogens through various immune responses [30–34], some of them mediated by members of the skin microbiome [35–39].

Disease resistance mediated by microbiomes can occur by direct and indirect defence mechanisms [18, 19]. Direct defence mechanisms take place between members of the microbiome and the invading pathogens and involve functional traits linked to i) competition for space and nutrients (e.g. biofilm formation [40] or quorum sensing [41, 42]), ii) active antagonism (e.g. contact-dependent inhibition by secretion systems [43, 44]), iii) biosynthesis of inhibitory metabolites (e.g. secondary metabolites [21, 45] or cell wall degrading enzymes [46, 47]). Indirect defence mechanisms refer to host responses triggered by microbiome members, and these include i) modification of the chemical properties of mucous barriers [48, 49] or ii) synthesis and release of immune effectors (cytokines, antimicrobial peptides or immunoglobulins [18, 50]).

In the case of amphibians, it has been shown that some members of the skin microbiome can contribute to disease resistance against Bd and Bsal through direct mechanisms such as biofilm formation [40, 51] or secondary metabolite biosynthesis [21, 45, 52]. However, until this day only a handful of anti-Bd secondary metabolites derived from amphibians have been fully characterized, and these include violacein [21], 2,4 DAPG [45], prodigiosin [53], tryptophol [54], and viscosine-like lipopeptides [55]. Even though the anti-Bd potential of amphibian skin microbes has been described in numerous amphibian species [36, 37, 40, 51, 53, 56, 57], there is little information about the genetic mechanisms involved in host protection against chytridiomycosis [22, 58].

A plethora of host-related [59–62], microhabitat related [63–68], and biogeographically related [69–72] factors influencing amphibian skin microbiome taxonomic diversity have been described. Moreover, it has been shown that differential health-disease outcomes against chytridiomycosis are linked to differences in microbiome community composition [63, 65, 73, 74]. Noteworthy, it has been suggested that the skin microbial composition is a good predictor for microbiome functional profiles in Bd susceptible amphibian species [22]. However, it remains unclear whether structure-function links are also occurring in other amphibian species with less susceptibility to chytridiomycosis.

We previously showed that skin bacterial communities of the endangered axolotl *Ambystoma altamirani* were influenced by seasonality and environmental variation across sampling locations, but not by Bd infection status [75]. We determined that *A. altamirani* skin microbiota was dominated by bacterial families with recognized anti-Bd activity [76] such as Burkholderiaceae, Chitinophagaceae and Pseudomonadaceae [75]. In addition, Bd infection dynamics in *A. altamirani* populations suggests that this amphibian species is tolerant to chytridiomycosis [77]. In this study, we applied gene-level metagenomics and constructed metagenome assembled genomes (MAGs) to describe functional genomic diversity of the *A. altamirani* skin microbiome. We evaluated the influence of seasonality, sites from distinct elevations and Bd infection status on the skin microbiome functional genomic diversity, including potential antifungal traits that could be important for host tolerance to chytridiomycosis.

We hypothesize that general functional traits of the *A. altamirani* skin microbiome would vary between consecutive seasons and among sites with distinct elevations (high-medium-low) in line with changes in the taxonomic diversity [75]. In addition, considering the tolerant status of *A. altamirani* against Bd [77], and the high abundance of potential anti-Bd bacteria over the skin of this axolotl species [75], we also hypothesize that potential antifungal traits of the skin microbiome should be prevalent across time and space despite functional variation of general functional traits.

## Methods

### Sample selection and sequencing procedures

In a previous study [75], skin swabs from metamorphic (without gills) and pre-metamorphic (with gills) *A. altamirani* axolotls were obtained across the four seasons of one year (from July 2019 to March 2020) from four different streams distributed along an altitudinal gradient. These samples were previously used for 16S metabarcoding [75] and Bd detection [77]. In this study, 40 samples from pre-metamorphic axolotls were selected for shotgun metagenome sequencing since pre-metamorphic axolotl bacterial communities were clearly influenced by seasonality and sites with different elevations [75]. Specifically, ten samples per season were selected, being five of them infected with Bd and five not infected. The only exception were the samples from autumn, which were eight Bd-infected samples and two not infected, due to limited amounts of DNA in some of the original samples (Table S1 in File S1). Samples were also chosen from different sites/streams with variable elevation ranging from 3087 metres above sea level (m.a.s.l) to 3447 m.a.s.l (Table S1 in File S1 and Fig. S1 in File S2). DNA extraction of the 40 samples was performed using the Qiagen DNeasy Blood and Tissue kit (Qiagen, Valencia, USA) and sent to CD Genomics (Shirley, NY, USA) for library construction and shotgun sequencing using the Illumina HiSeq 2×150 technology, at an average read depth of 20 million pair-end reads per sample.

### Sequence processing and construction of the *A. altamirani* skin microbiome gene catalog (AaSMGC)

Trim Galore [78] (V 0.6.2) was used for quality filtering and adapter trimming. Briefly, sequencing adapters were removed, and low-quality bases (Phred<20) were trimmed from each read. Then, host derived sequences and potential human contaminant sequences were removed from the whole data set by aligning sequenced reads against an index of the *A. mexicanum* (AmbMex60DD) and *Homo sapiens* (GRCh38) genome assemblies using Bowtie2 [79] (V 2.3.4.1, parameter: -sensitive). *A. mexicanum* (AmbMex60DD) genome was used as a reference to remove host reads because to date is the only available genome for any species of the genus *Ambystoma*.

Microbiome derived reads were recovered using Samtools [80] (V 1.9) and these were assembled using MegaHit [81] (V 1.1.3, parameter: --presets meta-sensitive -t 16 m 0.5). Then, the BBmap package [82] was used to recover contigs with a minimum length of ≥500 bp. Open reading frames (ORFs) were predicted for each contig using Prodigal [83] (V 2.6.3, parameter: -p meta). After discarding ORFs with a length <100 bp, the remaining ones were clustered using CD-HIT [84] (V 4.7, parameter: -c 0.95 n 8 G 0 -aS 0.9 g 1 -d 0) to generate a dereplicated gene catalogue referred here as *Ambystoma altamirani* Skin Microbiome Gene Catalogue (AaSMGC).

### Functional and taxonomic profiling of the AaSMGC

AaSMGC was functionally and taxonomically profiled using eggnog-mapper [85] (emapper-V 2.1.8, parameter: --cpu 40 m diamond --itype CDS –translate –evalue 0.00001 --sensmode very-sensitive) and the eggnog Orthologous Groups database (V 5.0.2). Results from eggnog-mapper were used to calculate the relative abundance of each gene in the AaSMGC. For this, quality filtered sequences for each sample were mapped against the AaSMGC using Bowtie2 (V 2.3.4.1, parameter ---very-sensitive). Reads that mapped to each gene entry in the AaSMGC were quantified using featureCounts [86] (V 2.0.1, parameter: -O -p -C -t CDS), these counts were used for diversity and differential abundance analysis.

### Gene richness and gene abundance profiles of the AaSMGC

Gene entries in the AaSMGC with a functional annotation were used to calculate gene diversity and differential abundance analysis. To evaluate the influence of seasonality, elevation/site, and Bd infection status over the functional genomic diversity of *A. altamirani* skin microbiome we discarded samples from Organillos site due to the low sample size (two samples from 3400 m.a.s.l). In this way, each elevation (low, medium, high) is represented by only one site. Before conducting gene diversity analyses gene counts obtained by feature counts were normalized using transcript (gene counts in this case) per million normalization (TPM) [87] to correct for differences in sampling depth between samples.

Kruskal-Wallis test followed by Dunn post-hoc comparisons were used to determine differences in gene richness between samples. To compare gene abundance profiles of *A. altamirani* skin microbiomes across seasons and site/stream elevations, a Bray-Curtis distance matrix was calculated followed by Permutational analysis of variance (PERMANOVA) and principal coordinate analysis (PCoA). In addition, the influence of Bd presence was evaluated between Bd infected and not infected axolotls collected within each season using a pairwise PERMANOVA test. *P*-values of pairwise PERMANOVA tests were adjusted for multiple comparisons using the Benjamini-Hochberg method (parameter: permutations=10 000, padj = ‘BH’) [88]. All statistical analyses were performed in R (V 4.2.3), using the packages Vegan [88] (V 2.6–4) and ecole [89] (V 4.2.3) in R (V 4.2.3).

### Gene enrichment analysis of general functions and potential antifungal traits in the AaSMGC

DESeq2 [90] was used to identify differentially enriched genes in the AaSMGC across seasons (consecutive seasons), elevations/sites (low, medium, high), and Bd infection status (positive/negative). To account for differences in sampling depth between samples, DESeq2 normalizes gene counts by calculating the geometric mean of each gene, differential enriched genes are then identified based on a negative binomial distribution of mean gene variance between samples [90].

Before running DESeq2, gene entries of the AaSMGC <10 read counts among all samples were filtered out [90, 91], resulting in a subset of 42 281 AaSMGC gene entries. Genes with FDR ≤0.01 and LogFold change ≥1 were considered as differentially enriched. FDR values for each gene were calculated using the ashr package [92] implemented in DESeq2.

To evaluate if genes linked to potential antifungal functions differed across seasons, elevations/sites and Bd infection status, a PERMANOVA test was used comparing abundances of genes linked to bacterial communication and competition traits (BCC: biofilm formation, quorum sensing and secretion systems) and potential antifungal functions (AF: secondary metabolite and chitinolytic enzyme biosynthesis). Selection of these gene categories was based on published literature suggesting that these functions may contribute to host protection against Bd [22, 40, 53, 58, 93, 94].

To evaluate the influence of Bd infection intensity over BCC and AF gene abundance, Pearson correlation tests were performed using the stats package (parameters: method=‘spearman’, adjust=‘BH’). Only Bd-positive samples were considered to calculate the correlations (22 out of 40 samples). Significant correlations were those with *p*-values<0.05 and *p*≥0.5 or **≤** −0.5.

### Reconstruction of metagenome assembled genomes (MAGs)

A binning approach was used to reconstruct metagenome assembled genomes (MAGs) from 40 individual assemblies. To recover the maximum amount of MAGs, samples obtained within each season under the same Bd infection status and from the same site were co-assembled (e.g. summer samples from not infected axolotls). Briefly, assembled contigs were binned using the MetaWRAP [95] bin module (V 1.3.0, parameter: -t 16 m 16 l 1500 --maxbin2 --metabat2 –concoct). All the recovered bins were refined using the MetaWRAP bin_refinement module (parameters: -t 30 c 50 x 10), completeness and contamination for each MAG were calculated using CheckM [96] (V 1.0.12), and only MAGs with completeness ≥50 % and ≤ 10 % contamination were retained. dRep [97] (V 3.4.0, parameter: -p 80 pa 0.90 -sa 0.99 -nc 0.30 cm larger --ignoreGenomeQuality) was used to obtain a set of not redundant MAGs with an ANI cutoff of 99 %, which resulted in the recovery of 50 not redundant MAGs.

Taxonomy was assigned to each not redundant MAG using GTDB-Tk [98] (V 2.1.1, parameter: classify_wf --extension fa --cpus 20). A maximum-likelihood tree was inferred using IQ-TREE [99] (V 2.1.4, parameter: -m LG+R4 T 30 -B 1000) using a nucleotide substitution model that was selected with IQ-TREE ModelFinder (parameter: -m MF -T 30) based on the multiple sequence alignment of GTDB-Tk BAC120 marker set. The prevalence and abundance of each recovered MAG on each sample were calculated as genome copies per million reads (GCPMR) using the quaint_bins module of MetaWrap.

### Inference of the potential antifungal functions on *A. altamirani* MAGs

MAGs were annotated to evaluate the presence of genes linked to BCC and AF functional traits. First ORFs were predicted from each MAGs using Prokka [100] (V 1.14.6, parameters: default) followed by functional annotation using eggnog-mapper (emapper-V 2.1.8, parameter: --cpu 40 m diamond --evalue 0.00001). The presence of BCC and AF functional traits was considered if at least one gene linked to these functions was present in the MAGs. In addition, to better understand the potential contribution to host defence against fungal pathogens, biosynthetic gene clusters (BGCs) were predicted for each MAG using antiSMASH [101] (V 6.1.1, parameters: --genefinding-tool none --fullhmmer --tigrfam --cc-mibig --cb-general --cb-knownclusters --cb-subclusters --asf --rre --pfam2go --smcog-trees). The identity of BGCs was evaluated according to ClustBlast [101] and MiBiG [102] repositories.

## Results

### Taxonomy and functions present on the *A. altamirani* skin microbiome

A total of 839.9 million pair-end reads were obtained from the 40 *A*. *altamiran*i skin microbiome samples. After quality filtering and host contamination removal, an average of 2 million quality pair-end reads were retained per sample ranging from 0.53 to 10.4 million reads (Table S1 in File S1). Then, quality-filtered reads were assembled into 494 430 contigs (Table S2 in File S1), and 1 098 389 open reading frames (ORFs) were predicted from the assembled contigs.

**Fig. 1.**
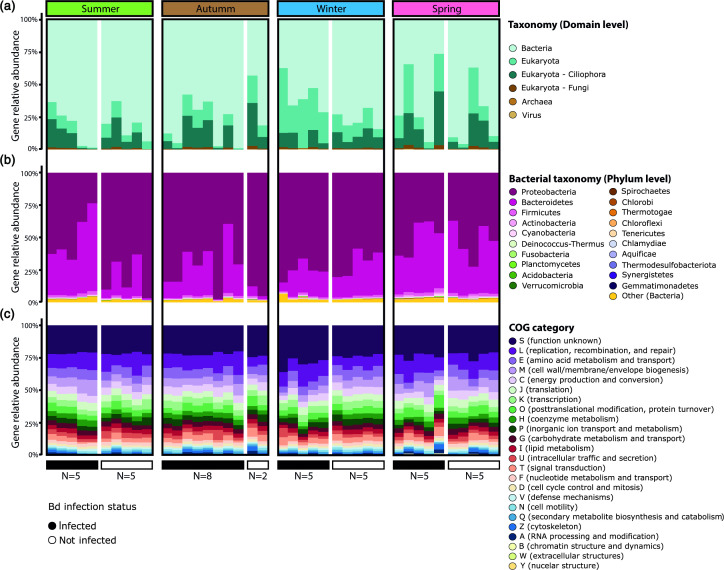
Relative abundance of gene entries in the AaSMGC. (**a**) Taxonomic relative abundance at the domain level. (**b**) Taxonomic relative abundance at the phylum level only for bacterially derived gene entries. (**c**) Functional relative abundance of gene entries based on COG categories. Each bar corresponds to a single sample and is grouped based on sampling season and Bd infection status.

After de-replication 190 932 unique ORFs were retained and 92 107 (48.24 %) of them were functionally and taxonomically annotated. The annotated genes comprised the *A. altamirani* skin microbiome gene catalogue (AaSMGC) that was used for further analyses. Gene entries of the AaSMGC were mostly derived from Bacteria (73.4 %) followed by Eukaryota (26.33 %), Viruses (0.158 %), and Archaea (0.0786 %) (Fig. 1a). Eukaryotes included protists and fungi among other microeukayotes, and a high proportion of them was classified as the phylum Ciliophora (11.5 %) while fungi represented only 0.004 % of the AaSMGC annotated genes (Fig. 1a). Noteworthy, our results showed that 94.5 % of bacteria genes in the AaSMGC were derived from the Proteobacteria (66.2 %) and Bacteroidetes (28.3 %) phyla (Fig. 1b).

Functional annotation of the AaSMGC showed 24 Clusters of Orthologous Genes (COG), being S (function unknown 23.5 %), L (replication, recombination and repair 11.8 %), E (amino acid transport and metabolism 7.12 %), M (cell wall or envelope biogenesis (6.4 %), and C (energy production and conversion (5.5 %) the most abundant COGs across all samples (Fig. 1c).

### Gene richness and gene abundances of the *A. altamirani* skin microbiome differ across seasons and elevations

Gene richness of the *A. altamirani* skin microbiome differed significantly across seasons (Kruskall-Wallis, Χ2=14.7, df=3, *p*-value=0.002), but post-hoc tests showed no significant differences between consecutive seasons (Table S3 in File S1 and Fig. S2A in File S2). Sites with distinct elevation showed significant differences in gene richness (Kruskall-Wallis, Χ2=7.3, df=2, *p*-value=0.02) and post-hoc tests indicated these differences were significant between high-medium and medium-low elevations (Table S3 in File S1 and Fig. S2B in File S2). No significant differences in gene richness were found between Bd infected and not infected samples (Wilcoxon, W=131, *p*-value=0.1) (Fig. S2C in File S2).

**Fig. 2.**
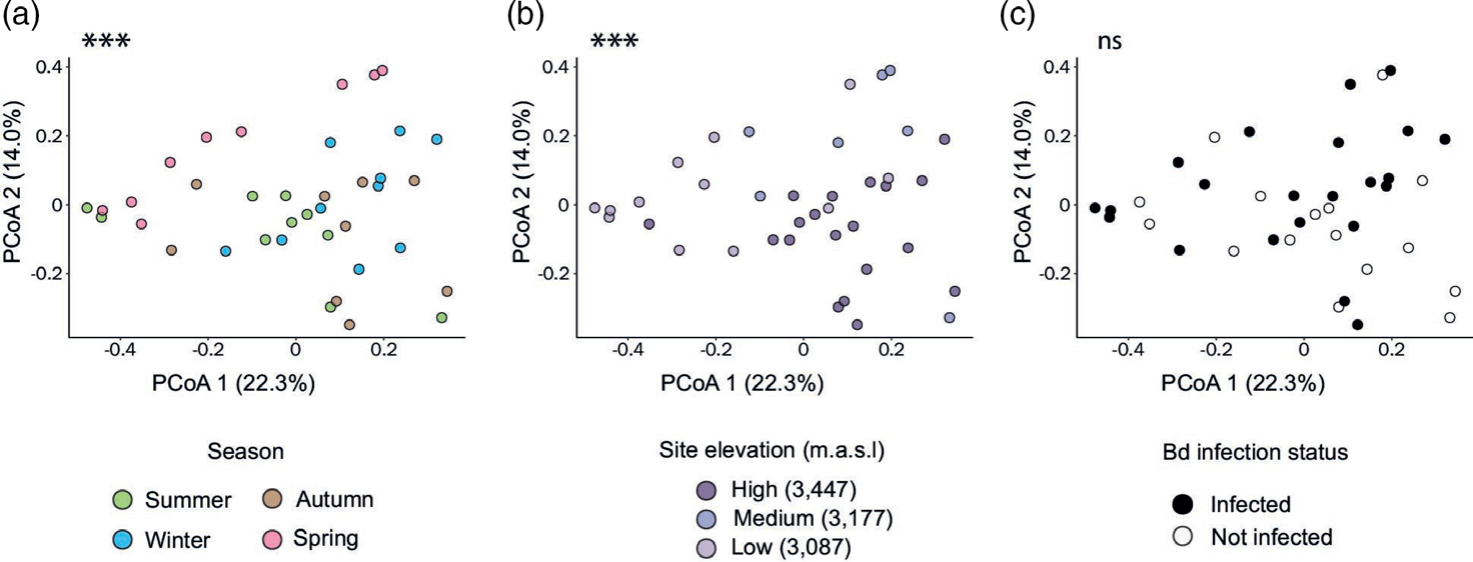
Principal coordinate analyses (PCoA) comparing gene abundance profiles of the *A. altamirani* skin microbiome based on Bray-Curtis distances: (a) across seasons, (**b**) across sites with distinct elevations and (c) between Bd infected and not infected axolotls. Asterisks indicate significant differences and ns indicates not significant differences.

In addition to gene richness, gene relative abundance profiles of the *A. altamirani* skin microbiome differed across seasons (PERMANOVA, R2=0.18, F=2.6, df=3, *p*-value=9.9e-5) (Fig. 2a) and elevations (PERMANOVA, R2=0.18, F=4.05, df=2, *p*-value=9e-5) (Fig. 2b). Pairwise PERMANOVA comparisons showed significant variation between consecutive seasons, except for the summer-autumn comparison (Table S4 in File S1), and between all elevation/site comparisons (Table S5 in File S1).

Gene abundance profiles did not significantly differ according to Bd infection status (PERMANOVA, R2=0.2, F=1, df=1, *p*-value=0.3) (Fig. 2c). Pairwise comparisons of Bd-infected and not infected samples within each season showed that gene abundances only differed in samples from the winter season (PERMANOVA, R2=0.6, F=12.4, *p*-value=0.008) (Table S6 in File S1). No significant differences were identified between Bd infected and not infected axolotls within sites with distinct elevation (Table S6 in File S1).

### Functional genomic changes of the *A. altamirani* skin microbiome across seasons, elevations and Bd infection status

**Fig. 3.**
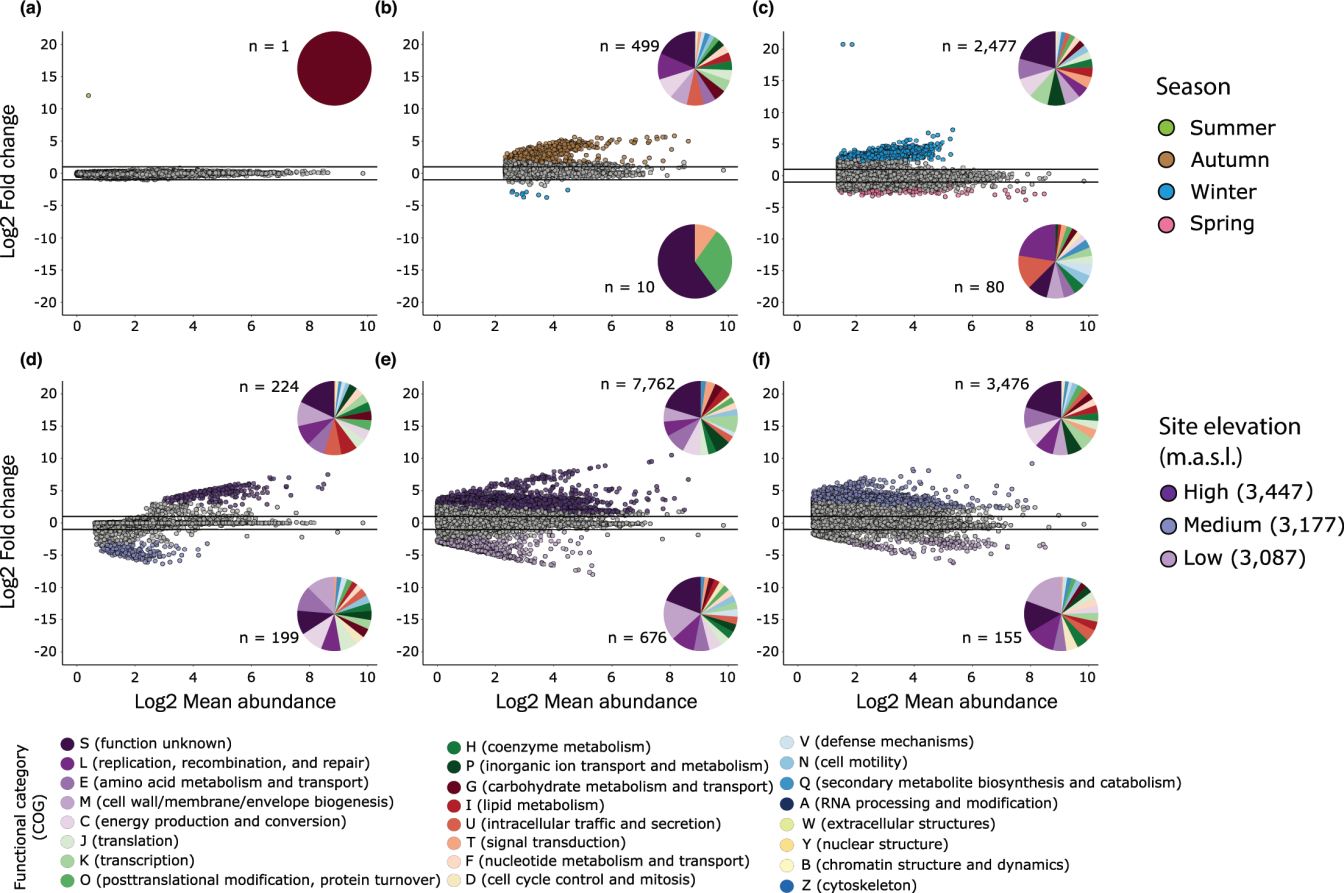
Differentially enriched genes across seasons (**a, b, c**) and elevations/sites (**d, e, f**) using DESeq2 and shown in MA plots. (**a**) Summer-autumn comparison. (**b**) Autumn-winter comparison. (**c**) Winter-spring comparison. (**d**) High-medium elevation comparison. (**e**) High-low elevation comparison. (**f**) Medium-low elevation comparison. Solid black lines represent the 1 Log2 Fold change threshold. Pie plots inside each panel depict the functional identity of the differentially enriched genes based on COG categories.

We explored changes in gene enrichment across seasons, elevations/sites and Bd infection status in the *A. altamirani* skin microbiome. Differentially abundance test (Deseq2) revealed that only one gene differed between the summer-autumn comparison (Fig. 3a). In contrast, 509 and 2557 genes were differentially enriched between autumn-winter and winter-spring seasons respectively. Specifically, 98 % (499 out of 509) of the enriched genes identified between autumn-winter seasons belonged to the autumn season (Fig. 3b). Meanwhile, 96 % (2477 out of 2557) of the enriched genes between winter-spring seasons belong to the winter season (Fig. 3c). Differentially enriched genes found across seasonal comparisons were mainly linked to the COG categories S (function unknown), L (replication, recombination and repair), E (amino acid transport and metabolism), M (cell wall/membrane/envelope biogenesis) and C (energy production and conversion).

A total of 423 (high-medium), 8438 (high-low), and 3631 (medium-low) genes were identified as differentially enriched between samples from different elevations/sites. In the case of the high-medium (Fig. 3d) comparison 53 % (224 out of 423) of the genes were enriched in high elevation samples and 47 % (199 out of 423) in medium elevation samples. Further, 92 % (7762 out of 8438) and 95.6 % (3476 out of 3631) of the enriched genes identified between high-low (Fig. 3e) and medium-low (Fig. 3f) were from high and medium elevations respectively. Differentially enriched genes were mainly linked to the COG categories S (function unknown), L (replication, recombination and repair), and M (cell wall/membrane/envelope biogenesis).

When comparing gene enrichment of Bd infected and not infected axolotls within each season we found that 1316 were differentially enriched within the winter season with 99.8 % (1314 out of 1316) of them being enriched in not infected axolotls (Fig. S3C in File S2). Only four and two genes were differentially enriched within samples from summer and autumn seasons respectively (Fig. S3A and B in File S2), while during spring season no differentially enriched genes were identified (Fig. S3D in File S2). Differential enriched genes identified within winter season were mainly classified under the S (function unknown), E (amino acid metabolism and transport), and C (energy production and conversion) COG categories (Fig. S3C in File S2).

### Potential antifungal functions of the *A. altamirani* skin microbiome are influenced by seasonality, elevation, and Bd presence

**Fig. 4.**
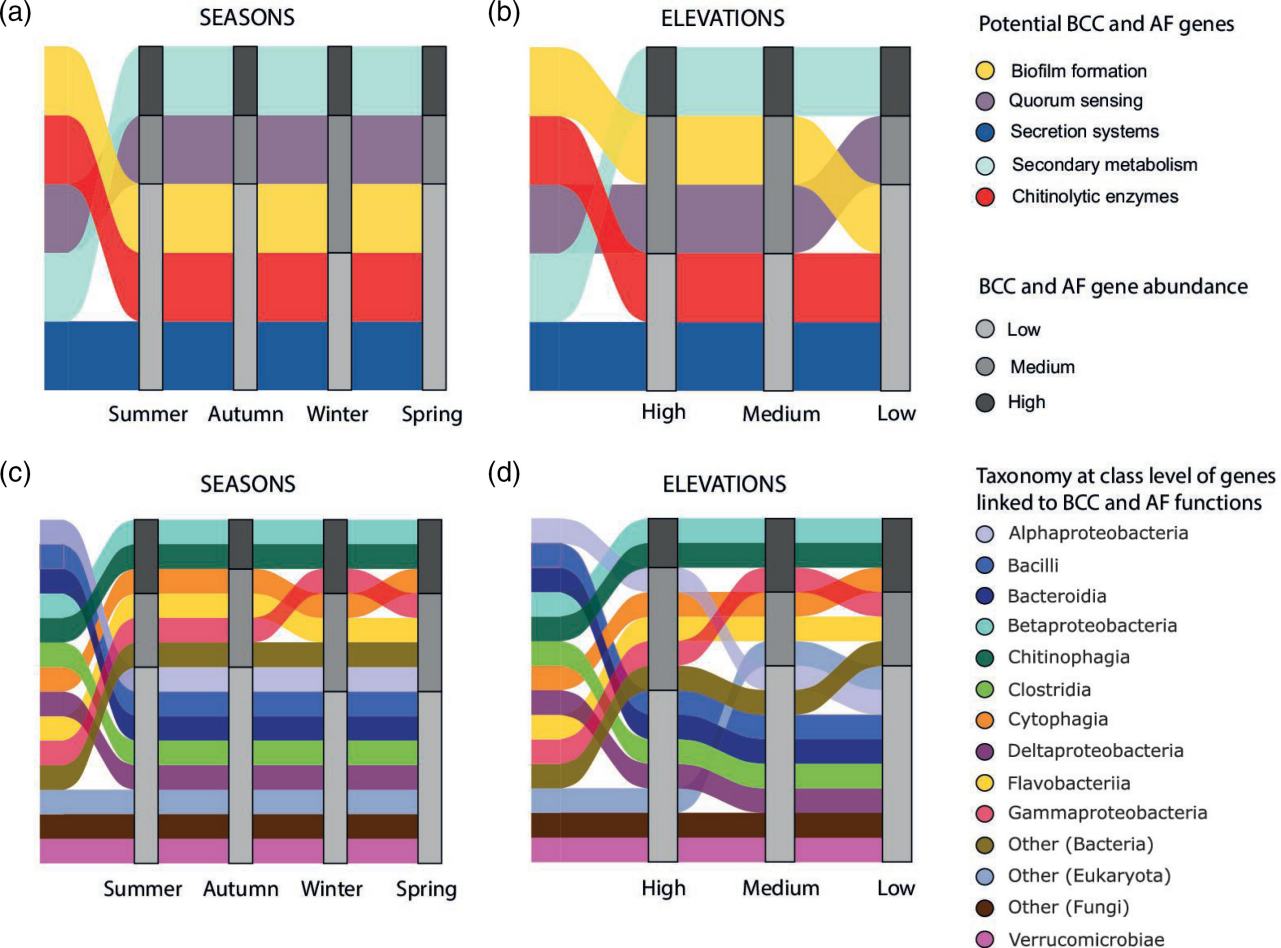
Changes in BCC and AF gene counts across (a) seasons and (b) elevations shown in alluvial plots. Taxonomic bacterial abundance at class level for the BCC and AF genes (c) between consecutive seasons and (d) across sites with distinct elevation. Bars in the alluvial plots coloured black-grey scale depict abundance levels for genes within each season or elevation range.

A total of 5196 genes in the AaSMGC were linked to BCC and AF, of these 72.8 % were linked to secondary metabolism, followed by quorum sensing (15.95 %), biofilm formation (8.8 %), secretion systems (1.67 %) and chitinolytic enzyme biosynthesis (0.71 %) (Fig. 4a, b). Noteworthy, 52.2 % of the BCC and AF genes were derived from the bacterial class Betaproteobacteria, followed by Chitinophagia (20.9 %), and Cytophagia (11.2 %) (Fig. 4c, d). In addition, our results showed that the gene abundance profiles of BCC and AF genes significantly differed across seasons (PERMANOVA, R2=0.19, F=2.7, df=3, *p*-value=9.9e-5) and elevations/sites (PERMANOVA, R2=0.18, F=3.8, df=2, *p*-value=9.9e-5). Pairwise comparisons showed that BCC and AF genes significantly differed between autumn-winter (PERMANOVA, R2=0.09, F=1.68, *p*-value=0.02) and winter-spring seasons (PERMANOVA, R2=0.19, F=4, *p*-value=0.001) (Table S7 in File S1). In addition, gene abundance profiles of the BCC and AF genes significantly differed between all elevations (Table S8 in File S1).

We found that BCC and AF genes significantly differed between Bd infected and not infected axolotls only during the winter season (PERMANOVA, R=0.18, F=1.8, *p*-value=0.008) (Table S9 in File S1). In addition, Spearman’s correlation test showed that 244 genes had significant correlations with Bd infection intensity (Fig. S4 in File S2). Specifically, 93.4 % (228 out of 244) of these genes were negatively correlated with Bd infection intensity levels and the other 6.6 % (16 out of 244) showed positive correlations (Table S10 in File S1). Genes linked to secondary metabolism, quorum sensing and secretion systems exhibited both positive and negative correlations, however only negative correlations were identified for genes linked to biofilm formation (Table S10 in File S1).

### MAGs from the *A. altamirani* skin microbiome are taxonomically diverse and prevalent across seasons and sites with distinct elevations

To better describe the possible contribution of specific bacterial groups in host protection against pathogens, we obtained metagenome assembled genomes (MAGs) from the *A. altamirani* skin microbiome. After binning and MAG refinement, we recovered 151 MAGs with ≥50 % completeness and ≤10 % contamination. After dereplication, the initial set of 151 MAGs was reduced to 50 not redundant MAGs. These ranged from 0.76 Mb to 6.8 Mb, with N50 values ranging from 2.1 Kb to 271.5 Kb (Table S11 in File S1).

**Fig. 5.**
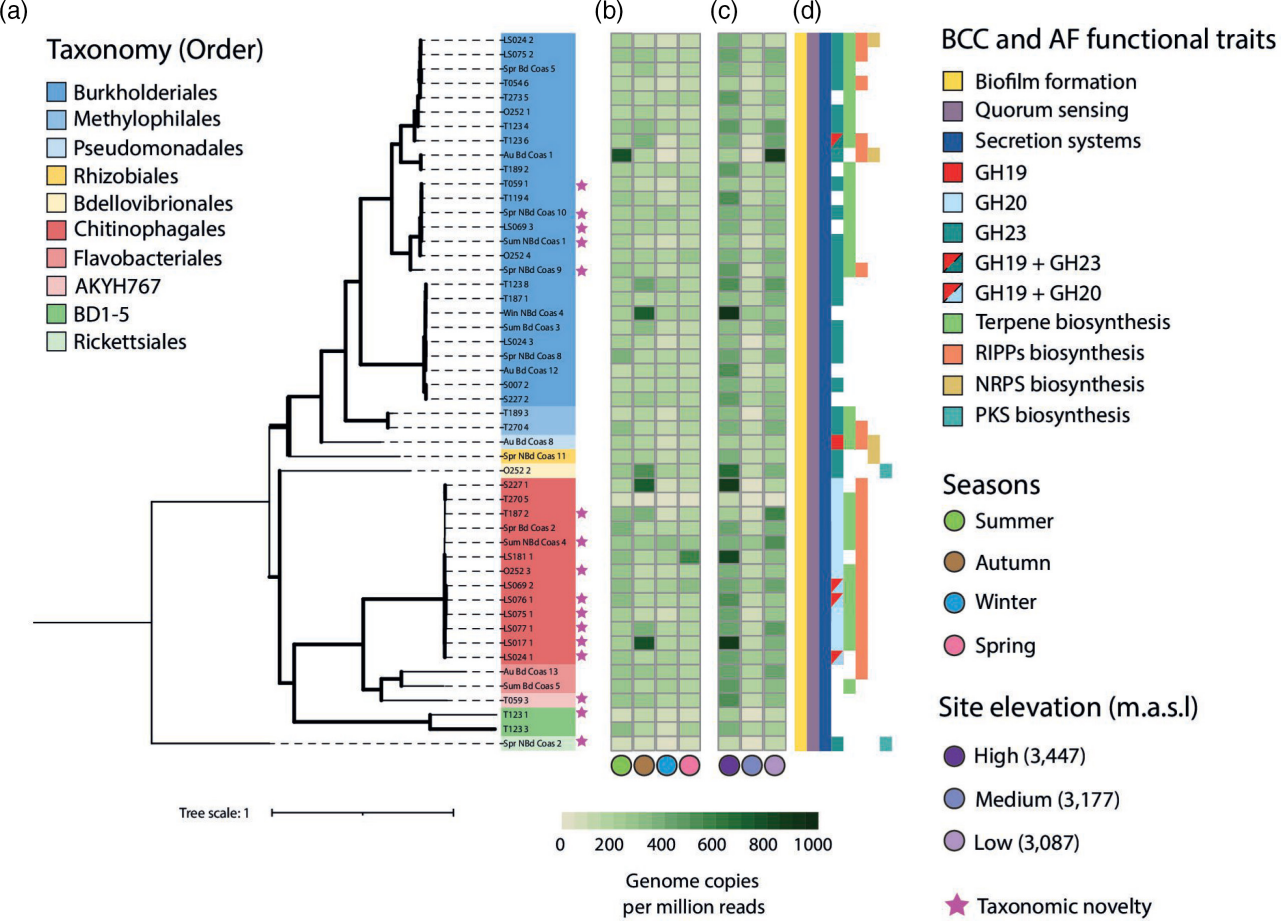
MAG taxonomy, abundance across season and elevations, and presence of BCC and AF functional traits. (**a**) Maximum likelihood tree of the 50 *A*. *altamirani* skin microbiome derived MAGs based on GTDB-Tk taxonomic classification. Pink stars aside tree tips depict MAGs classified as taxonomic novelties. Summarized MAG abundance as genome copies per million reads (GCPMR) (**b**) across seasons and (**c**) across sites with distinct elevation. (**d**) BCC and AF functional traits present on each MAG (represented by at least one gene). Bold lines in the maximum likelihood tree represent branches with bootstrap support >70.

The 50 MAGs recovered from the *A. altamirani* skin microbiome were classified into four different bacterial phyla (Bacteroidetes, Bdellovibrionota, Patescibacteria, and Proteobacteria) and ten bacterial orders (Fig. 5a). According to GTDB-Tk classification criteria based on the relative evolutionary divergence (RED) of MAGs, 16 of these MAGs were identified as taxonomic novelties. Novel MAGs were classified at order level as Chitinophagales (eight MAGs), AKYH767 (one MAG, within the phylum Bacteroidetes), Burkholderiales (five MAGs), Rickettsiales (one MAG), and BD1-5 (one MAG, within the phylum Patesibacteria) (Fig. 5a).

A total of 43 286 genome copies per million reads (GCPMR) from the 50 non-redundant MAGs were found across the 40 skin microbiome samples. Burkholderiales (23 904 GCPMR) and Chitinophagales (13 319 GCPMR) MAGs were the most abundant accounting for 85 % of the relative abundance of MAGs across seasons and elevations (Fig. 5b, c). Moreover, we found that 48 MAGs were prevalent across seasons (Fig. 5b, Table S12 in File S1) and elevations (Fig. 5c, Table S13 in File S1), with the exception of two of them which were not found during the autumn season and in the medium elevation site (Fig. 5b, c, Table S12 and S13 in File S1).

### Bacterial competition, communication (BCC) and antifungal (AF) genes are widespread in *A. altamirani* derived MAGs

Genes linked to BCC and AF functions were identified at least once in all the 50 recovered MAGs (Fig. 5d, Table S1-S50 in File S3). Noteworthy, genes linked to chitinolytic enzyme biosynthesis were identified in MAGs from the bacterial orders Burkholderiales and Chitinophagales, and they were annotated as glycoside hydrolases from the GH19, GH20, and GH23 families. Specifically, GH19 and GH23 are recognized as chitinolytic enzymes, while the GH20 family is recognized as N-acetylglucosaminidases.

Biosynthetic gene clusters (BGCs) were predicted for each MAG using antiSMASH (Table S14 in File S1). Our results showed that BGCs were present in 40 of the 50 MAGs recovered from the *A. altamirani* skin microbiome (Fig. 5d). Specifically, we identified 79 BGCs (Table S14 in File S1), and most of them were linked to terpene biosynthesis (34 out of 79) and ribosomally synthesized and post-translationally modified peptides (RiPP, 27 out of 78) (Fig. 5d). Noteworthy 63 (out of 79) of the predicted BGCs were identified in Burkholderiales (36 BGCs), and Chitinophagales (27 BGCs) MAGs (Fig. 5d).

## Discussion

Several studies have shown that multiple biotic and abiotic factors are influencing community composition and taxonomic diversity of amphibian skin microbiomes [64, 68, 74, 103]. However, little is known about the influence of these factors over the functional repertoire of amphibian skin microbiomes. A recent study showed that skin microbial taxonomic composition was a good predictor of functionality in the skin microbiome of an amphibian species susceptible to chytridiomycosis [22]. However, it remains unclear whether these observations are a common trend for all amphibian species.

In this work, we described the functional genomic diversity of the *A. altamirani* skin microbiome and evaluated whether general gene functions and potential antifungal traits varied across time (seasons), space (sites with distinct elevations), and Bd infection status. For this, we compiled a gene catalogue (AaSMGC) composed of unique annotated genes to describe functional genomic variation and to identify genes linked to BCC and AF traits, which could be important for host defence against Bd. Lastly, we explored the potential contribution of specific bacterial taxa in host protection against Bd through the assembly of 50 MAGs recovered from *A. altamirani* skin microbiome and the search for BCC and AF traits within them.

We previously showed that bacterial community composition and taxonomic diversity of *A. altamirani* was influenced by environmental variation across seasons and sampling locations (from distinct elevations) [75]. In this work, we demonstrated that the functional genomic richness and abundance of *A. altamirani* skin microbiome was influenced by seasonality and sites with distinct elevations in the same way as taxonomic diversity [75], suggesting that skin microbiomes have a dynamic response to environmental variation and other site-specific factors. Our results fall in line with [22, 22] observations about the potential predictive relation between taxonomic diversity and microbiome functional traits, when analysing general gene profiles.

Previous studies have explored whether the presence and infection dynamic of the pathogen Bd is linked to changes in skin microbial diversity and structure [63, 64, 66, 70, 73, 104, 105]. Evidence suggests that Bd presence has contrasting effects over amphibian skin microbiomes; in some species the presence of the pathogen correlated with changes in skin community composition [66, 73], while in other cases, no differences were described between infected and not infected hosts [75, 104].

Previously we showed that Bd infection status did not influence *A. altamirani* skin bacterial taxonomic composition, however we did find that specific ASVs exhibited positive and negative correlations with Bd infection loads [75]. In this study we showed that gene richness and abundance profiles of the general and potential antifungal traits of the *A. altamirani* skin microbiome did not differ between infected and not infected axolotls, with the exception of samples collected during the winter season. This finding may be linked to a possible interaction between the pathogen’s biology and the protective role of the microbiome during particular environmental conditions (winter) in these populations. In this respect, studies have shown that Bd infection prevalence [106, 107] and virulence [108, 109] are modulated by temperature [110–112] and we have previously found that prevalence and infection intensity in *A. altamirani* populations was modulated by environmental factors [77].

The antifungal role of amphibian skin microbiomes has been described in various amphibian species [38, 52, 56, 57, 73], but very little is known about the genetic mechanisms responsible for the anti-Bd capacity of these microbiomes. To date, only a handful of BCC (biofilm formation [40], quorum sensing [113, 114], and secretion systems [58]) and AF (biosynthesis of secondary metabolites [53, 58, 108] and chitin degradation [22]) functional traits have been linked to potential host protection against Bd. In this study we described the presence of several genes linked to potential antifungal traits in both the AaSMGC and in the recovered MAGs. Moreover, we found that genes linked to secondary metabolite production quorum sensing and biofilm formation were negatively correlated with Bd infection intensity. These finding may support previous findings about the protective role of biofilm formation against chytrid pathogens [40].

Noteworthy, BCC and AF genes of the AaSMGC were mainly derived from the Proteobacteria and Bacteroidetes phyla, suggesting that these taxonomic groups may play an important role in host protection against pathogens as seen in *in vitro* assays [45, 51, 52, 56, 57]. Furthermore, we showed that BCC and AF genes are present in the whole set of recovered MAGs and that most of these MAGs are widespread and prevalent across seasons and elevations. Noteworthy most of these genes were identified in Burkholderiales (Proteobacteria) and Chitinophagales (Bacteroidetes) MAGs. Bacterial members of these taxa have been previously reported as the most prevalent bacterial groups of the *A. altamirani* skin microbiome [75]. Moreover, as bacteria from these orders are recognized as anti-Bd bacteria [56, 57, 76] it could be possible that the high abundance of these bacterial groups are linked to their role in modulating fungal abundances, as previously demonstrated in other amphibian species [115].

Genomic analysis of bacteria isolated from amphibian skin revealed the presence of genes linked to biosynthesis of various metabolites that could be linked to host protection against Bd and host-microbiome interactions [93, 94, 116]. Specifically, genes linked to AF traits have been linked to the biosynthesis of chitinolytic enzymes [22], and secondary metabolites such as ribosomal and non-ribosomal peptides (RiPP and NRPS), aryl-polyenes, polyketides, or bacteriocins [58, 93, 94].

Among the AF functional traits identified in this study, genes linked to chitinolytic enzyme biosynthesis and BGCs for terpene and RIPP biosynthesis were the most prevalent in Burkholderiales and Chitinophagales MAGs. Specifically, chitinolytic enzyme biosynthesis has been recognized as a common defence mechanism [46, 117–119] employed by bacteria, plants, animals, and fungi [120–122] to inhibit the growth of fungi. Moreover, it has been shown that amphibians exposed to Bd exhibit an increased abundance of a bacterial-derived chitin deacetylase [22], an enzyme that contributes to chitin degradation [123]. Here we reported the presence of genes linked to chitin degradation in Burkholderiales and Chitinophagales MAGs including glycoside hydrolases from the GH19 [124], GH20 [125, 126], and GH23 [117, 127, 128] families. Noteworthy, GH23 enzymes were mainly identified in Burkholderiales MAGs, while GH20 enzymes were identified only in Chitinophagales MAGs suggesting these taxa may use different/complementary strategies for chitin degradation.

BGCs identified in Burkholderiales and Chitinophagles MAGs were mainly associated with terpene and RiPP biosynthesis. Terpenes are a vast group of chemical compounds produced by a wide range of organisms from bacteria to plants [129–131], these metabolites have diverse ecological functions related to environmental stress responses [132], host-microbial communication [133, 134], and microbial-microbial competition [120, 130, 135]. Genes linked to terpene biosynthesis have been identified in amphibian skin microbiomes [58, 136] and in bacterial genomes isolated from the amphibian skin [137]. Moreover it has been suggested that terpenes synthetized by skin bacteria could act as sex specific scents for amphibian hosts [138]. Further work is needed to elucidate if terpenes produced by amphibian skin bacteria are involved in host protection against Bd. Finally, RiPPs are a wide group of peptides implicated in microbial-microbial interactions [139, 140]. Given that nearly half of the RiPP clusters predicted in this study were linked to bacteriocin biosynthesis, we could suggest that these molecules are playing a role in bacteria-bacteria interactions [137–139, 141–143] within the skin microbiomes of *A. altamirani*.

## Conclusions

Overall, our results indicate that the functional genomic diversity of the *A. altamirani* microbiome varies across time and space (seasons and sites with distinct elevation), suggesting that taxonomic variation [75] is directly linked to functional variation in this system. Moreover, we identified genes linked to BCC and AF functions in all the recovered MAGs, suggesting that potential antifungal functions are a widespread and prevalent trait in the skin microbiome of *A. altamirani* and likely contribute to host tolerance against Bd infection. Further exploration of the anti-Bd activity of terpenes and chitinolytic enzymes would contribute to a better understanding of the role of microbiomes in host defence against chytridiomycosis.

## Supplementary Data

Supplementary material 1

Supplementary material 2

Supplementary material 3
