# Supplementary material for: Gene functions of the Ambystoma altamirani skin microbiome vary across space and time but potential antifungal genes are widespread and prevalent
Source: Microb Genom. 2024 Jan 19;10(1):001181. doi: 10.1099/mgen.0.001181 (PMC10868611; doi:10.1099/mgen.0.001181)

Supplementary File 2

Supplementary Figures

Gene functions of the *Ambystoma altamirani* skin microbiome vary across space and time but potential antifungal genes are widespread and prevalent

Emanuel Martínez-Ugalde[1,] Víctor Ávila-Akerberg[2], Tanya M. González Martínez[3] and Eria A. Rebollar[1*].

[1]Centro de Ciencias Genómicas, Universidad Nacional Autónoma de México. [2]Instituto de Ciencias Agropecuarias y Rurales, Universidad Autónoma del Estado de México, Toluca, Mexico. [3]Facultad de Ciencias, Universidad Nacional Autónoma de México, Mexico City, Mexico.

Figure S1. Map representing the sampling locations (red dots) where the *Ambystoma altamirani* samples were collected. Turquoise lines represent streams runoffs at Sierra de Cruces. Isidro Fabela and Jilotzingo municipal limits are shown in yellow lines.

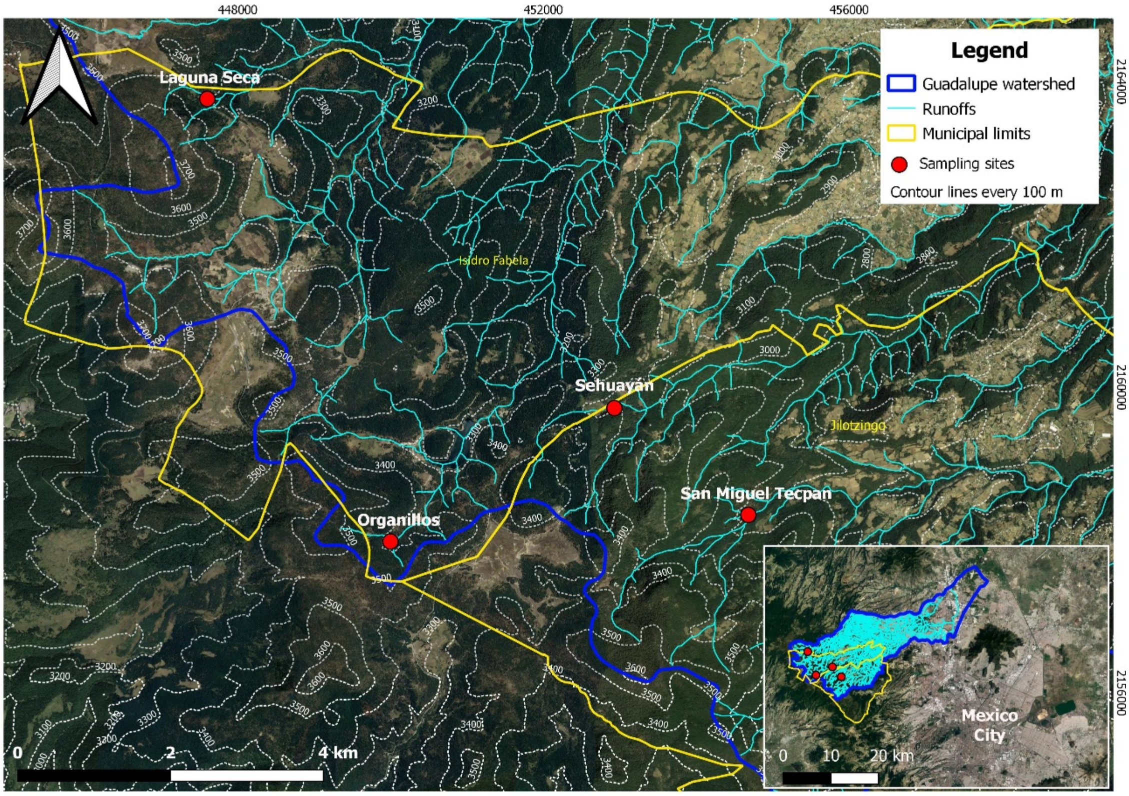

Figure S2. Gene richness of the *A. altamirani* skin microbiome across seasons, elevation/sites and Bd infection status. A) Gene richness comparisons across seasons. B) Gene richness comparisons across sites with distinct elevations. C) Gene richness comparisons between infected and not infected axolotls.

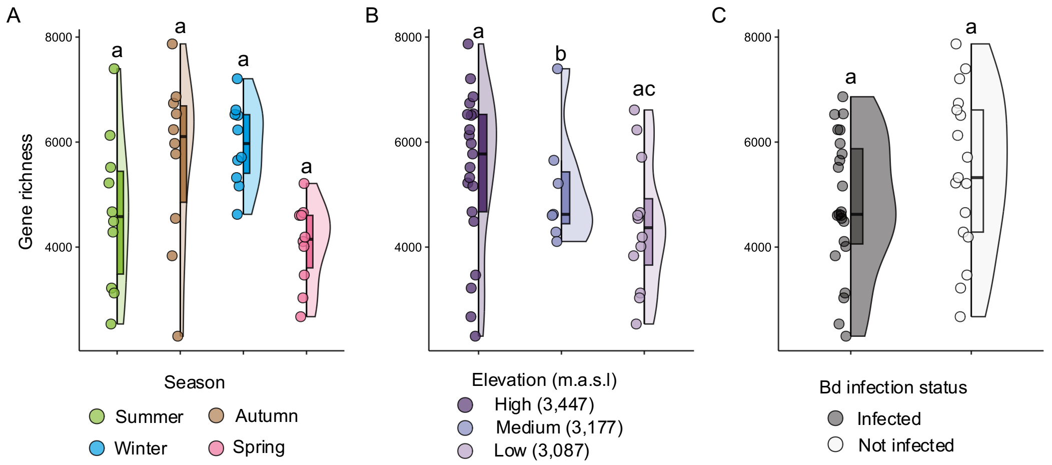

Figure S3. Differentially enriched genes between Bd infected and not infected axolotls within each season using DESeq2 and shown in MA plots. A) Summer season, B) autumn, C) winter, D) spring. Solid black lines represent the 1 Log2 Fold change threshold. Pie plots inside each panel depict the functional identity of the differentially enriched genes based on COG categories.

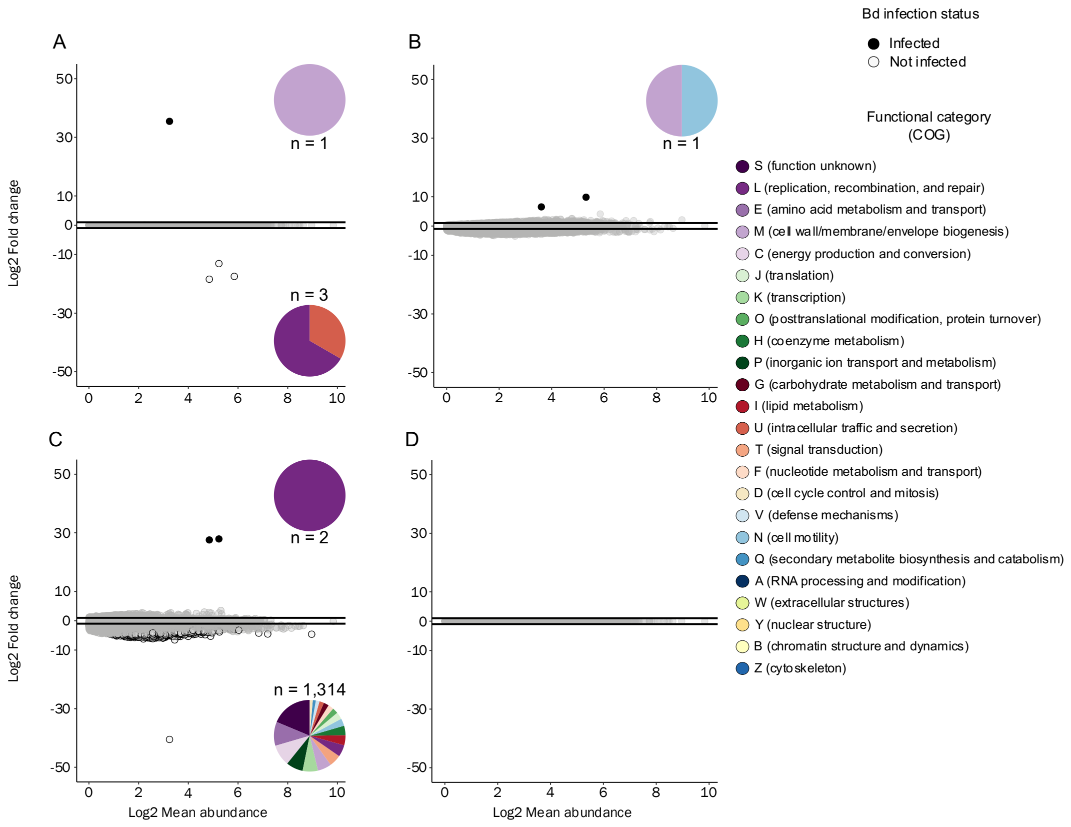

Figure S4. Spearman correlation tests between Bd infection intensity and potential antifungal (BCC and AF) gene abundances. Y axis shows Spearman's ρ correlation coefficient, X axis shows gene abundance as TPM (transcripts per million which in this case is gene counts). Circles in the plot are color coded by potential BCC and AF functional trait.

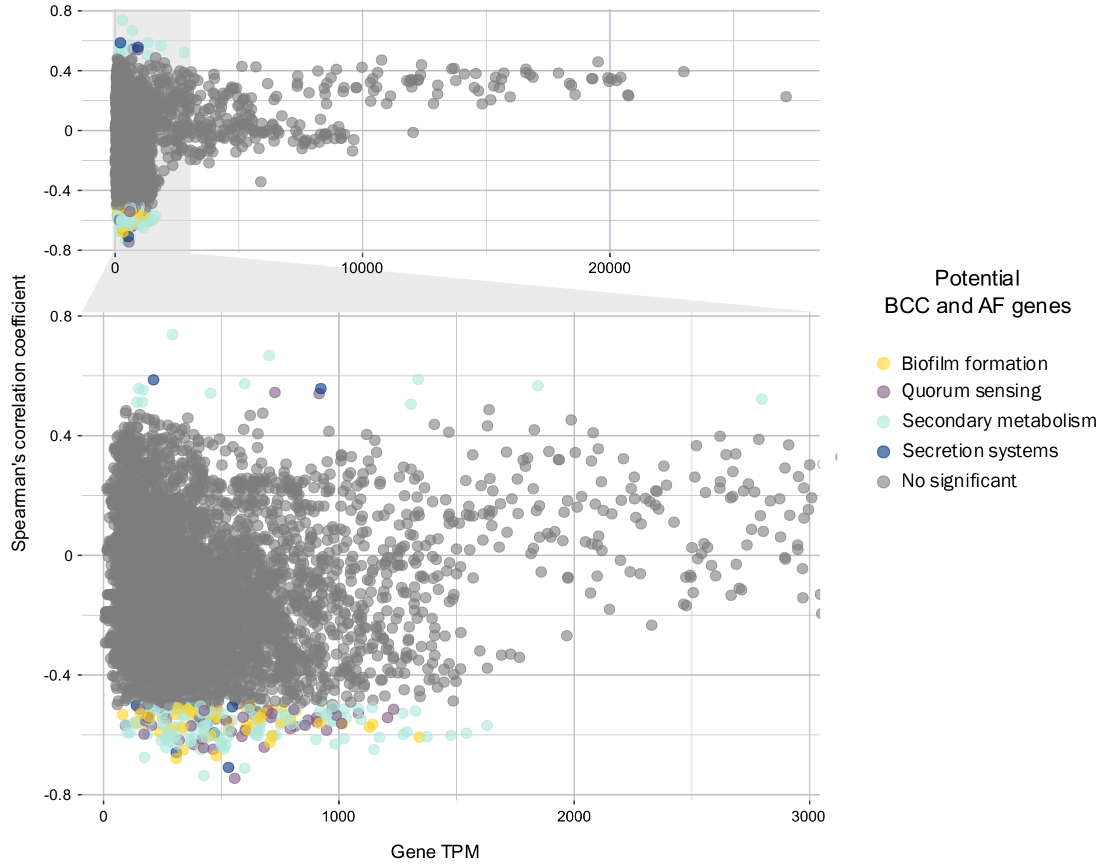

Supplement: Supplementary material 1 [file mgen-10-1181-s001.pdf]
